# Skeletal Muscle mRNA Splicing Variants Association With Four Different Fitness and Energetic Measures in the GESTALT Study

**DOI:** 10.1002/jcsm.13603

**Published:** 2024-12-02

**Authors:** Stefano Donega, Nirad Banskota, Esha Gupta, Marta Gonzalez‐Freire, Ann Zenobia Moore, Ceereena Ubaida‐Mohien, Rachel Munk, Linda Zukley, Yulan Piao, Chris Bergeron, Jan Bergeron, Arsun Bektas, Marta Zampino, Carole Stagg, Fred Indig, Lisa M. Hartnell, Mary Kaileh, Kenneth Fishbein, Richard G. Spencer, Myriam Gorospe, Supriyo De, Josephine M. Egan, Ranjan Sen, Luigi Ferrucci

**Affiliations:** ^1^ Longitudinal Studies Section (LSS) National Institute on Aging (NIA), National Institutes of Health (NIH) Baltimore Maryland USA; ^2^ Laboratory of Genetics and Genomics (LGG) National Institute on Aging (NIA), National Institutes of Health (NIH) Baltimore Maryland USA; ^3^ Translational Research in Aging and Longevity Group (TRIAL group) Fundació Institut d'Investigació Sanitària Illes Balears (IdISBa) Palma de Mallorca Spain; ^4^ Faculty of Experimental Sciences Universidad Francisco de Vitoria (UFV) Madrid Spain; ^5^ Clinical Research Core (CRC) National Institute on Aging (NIA), National Institutes of Health (NIH) Baltimore Maryland USA; ^6^ Confocal Imaging Facility National Institute on Aging (NIA), National Institutes of Health (NIH) Baltimore Maryland USA; ^7^ Laboratory of Clinical Investigation National Institute on Aging (NIA), National Institutes of Health (NIH) Baltimore Maryland USA; ^8^ Laboratory of Molecular Biology and Immunology (LMBI) National Institute on Aging (NIA), National Institutes of Health (NIH) Baltimore Maryland USA

**Keywords:** aging, alternative splicing, energy, exercise, kPCr, mitochondria respirometry, muscle, physical activity, VO_2_

## Abstract

**Background:**

Physical activity is essential for maintaining muscle mitochondrial function and aerobic capacity. The molecular mechanisms underlying such protective effects are incompletely understood, in part because it is difficult to separate the effects of disease status and physical activity. We explored the association of human skeletal muscle transcriptomic with four measures of energetics and mitochondria oxidative capacity in healthy individuals.

**Methods:**

Using RNA sequencing of vastus lateralis muscle biopsies from 82 GESTALT participants (52 males, aged 22–89 years), we explored gene and splicing variant expression profiles associated with self‐reported physical activity, peak oxygen consumption (VO_2_ peak), muscle oxidative capacity (kPCr) and mitochondrial respiration (Mit‐O_2_ flux). The effect of aging on gene expression was examined in participants with low and high VO_2_ peak.

**Results:**

The four measures of energetics were negative correlated with age and generally intercorrelated. We identified protein‐coding genes associated with four energetic measures adjusting for age, muscle fiber‐ratio, sex and batch effect. Mitochondrial pathways were overrepresented across all energetic variables, albeit with little overlap at the gene level. Alternative spliced transcript isoforms associated with energetics were primarily enriched for cytoplasmic ribonucleoprotein granules. The splicing pathway was up‐regulated with aging in low but not in high fitness participants, and transcript isoforms detected in the low fitness group pertain to processes such as cell cycle regulation, RNA/protein localization, nuclear transport and catabolism.

**Conclusions:**

A consistent mitochondrial signature emerged across all energetic measures. Alternative splicing was enhanced in older, low fitness participants supporting the energy‐splicing axis hypothesis. The identified splicing variants were enriched in pathways involving the accumulation of ribonucleoproteins in cytoplasmic granules, whose function remains unclear. Further research is needed to understand the function of these proteoforms in promoting adaptation to low energy availability.

## 1 Introduction

Physical activity is associated with higher skeletal muscle mitochondrial oxidative capacity [1, 2, 3]. Individuals who engage in regular physical activity exhibit higher levels of mitochondrial proteins and lower levels of splicing‐related proteins in their skeletal muscle [4, 5]. Octogenarian master athletes have higher muscle mitochondrial protein content and higher mitochondrial DNA (mtDNA) copy number compared to non‐athlete age/sex‐matched controls, along with less spliceosome proteins [3]. These findings led to the Energy‐Splicing resilience axis of aging posing that increased production of specific protein splicing variants occurs in response to low cell energy availability [6]. In support of this hypothesis, mitochondrial oxidative capacity assessed by Phosphorus‐31 (^31^P) Nuclear Magnetic Resonance (NMR) is associated overexpression of major spliceosome proteins [7] and mRNA isoforms that change significantly with aging pertain to proteins involved in oxidative phosphorylation [8].

No prior research comprehensively examined the skeletal muscle transcriptome as a function of distinct energy and fitness measures in the same individuals. Previous studies of gene expression associated with activity or fitness were conducted in model organisms, or in few individuals heterogeneous for health status [9]. Due to the interconnection between health and fitness, it is challenging to differentiate the effects of disease status from those attributable to physical activity.

We performed RNA sequencing in skeletal muscle biopsies from 50 men and 32 women who were screened to be extremely healthy. Using these data, we identified protein‐coding mRNAs, splicing variants and biological pathways associated with physical activity, cardiovascular fitness (peak oxygen consumption assessed by treadmill stress testing), muscle oxidative capacity (assessed by ^31^P‐Magnetic Resonance Spectroscopy, MRS) and mitochondrial respirometry (measured from permeabilized muscle fibers). Self‐reported levels of physical activity (PA) depend on cardiovascular fitness but also on attitude and individuals' choices. Maximum oxygen consumption during peak exercise (VO_2_ peak) is a measure of global fitness that results from combined effects of mitochondrial function, perfusion, and cardiopulmonary function on whole body exercise performance. Post‐exercise phosphocreatine recovery rate (kPCr) estimates oxidative capacity and depends on both intrinsic mitochondrial respiratory capacity and availability of oxygen and nutrients through muscle perfusion. Ex‐vivo Mitochondrial Oxygen Flux Respiration (Mit‐O_2_ flux) is a direct measure of intrinsic mitochondrial oxidative capacity. We expected these measures to only partially correlate since they tap into different domains of energetics.

We aim to detect transcriptomic signatures across a gradient of different energetic fitness measures, and we posited that differentially expressed genes, mRNA isoforms and enriched pathways linked to these energetic outcomes, would exhibit both overlaps and differences. Results of these analyses may offer insights into the biological mechanisms connecting adaptive muscle strategies to varying levels of fitness and the aging process.

## 2 Methods

### 2.1 Population Study

GESTALT is a longitudinal study conducted by the National Institute on Aging's (NIA) Intramural Research Program (IRP) in Baltimore, MD, USA. The study enrolled 50 men and 32 women older than 20 years free of major diseases, no active cancer within 10 years, no physical or cognitive impairments and minimal medication use. Participants were not professional athletes with a body mass index (BMI) of less than 30 kg/m^2^. Participants underwent a two‐day clinical visit to detect exclusion criteria and their demographic, physical and energetic parameters were evaluated. BMI was assessed as weight in kilograms divided by the square of the height in meters, and the obesity index was computed as the waist circumference to height ratio. The research protocol was approved by the Intramural Research Program of the US National Institute on Aging, and informed consent was obtained from each participant.

### 2.2 Energetic Fitness Measures

#### 2.2.1 Physical Activity

A modified Minnesota Leisure Time Physical Activity questionnaire was used to estimate physical activity [10]. Physical activity was operationally defined as the total self‐reported minutes of walking at a brisk/vigorous pace, and other vigorous activities, such as cycling, swimming, running, soccer, basketball, volleyball, aerobics, racquet sports, rowing or cross‐country skiing.

#### 2.2.2 Peak of Oxygen Consumption (VO_2_ Peak)

Maximum oxygen consumption during peak exercise (VO_2_ peak) was assessed by a modified version of the treadmill Balke protocol [11]. Concentrations of expired CO_2_ and consumed O_2_ were assessed with a gas exchange analyser (Ultima C2, MedGraphics, St. Paul, MN, USA). Oxygen consumption (mL/kg/min) was calculated every 30 s, and the highest value was recorded as the VO_2_ peak. The treadmill incline was progressively increased by 3% after each stage until voluntary exhaustion, with the first incline change at 45 s and subsequent increases every 3 min until steady state.

#### 2.2.3 Post‐Exercise Phosphocreatine Recovery Rate (kPCr)

The post‐exercise bioenergetic recovery rate in the lateral quadriceps muscle was estimated with ^31^P‐MRS, performed using a 3T Philips Achieva MR scanner (Philips, Best, Netherlands) [12, 13]. Participants positioned supine within the scanner performed repeated rapid forceful ballistic knee extensions. A sequence of ^31^P‐MRS spectra was acquired using a 10‐cm ^31^P‐tuned flat surface coil (PulseTeq, Surrey, UK) secured over the vastus lateralis muscle of the left thigh. Exercise was stopped when the height of the phosphocreatine (PCr) spectral line diminished to 33%–67% of baseline. The postexercise PCr recovery rate was calculated by fitting the time‐dependent changes in PCr peak area after exercise to a mono‐exponential recovery function using jMRUI (version 5.0) followed by quantification using AMARES [14]. The kPCr, or PCr recovery rate constant, was then determined as 1/τPCr, where τPCr is the PCr exponential recovery time constant [12].

#### 2.2.4 Ex‐Vivo Mitochondrial Oxygen Flux Respiration (Mit‐O_2_ Flux)

We conducted ex vivo mitochondrial respirometry measurements on 54 skeletal muscle biopsies obtained by a slightly modified version of the Bergström needle method [15]. A muscle tissue specimen (~15 mg) was cleared of adipose and connective tissue, then muscle fiber bundles were permeabilized in a saponin‐based solution and wet sample weight was measured for data normalization. Oxygen flux across ADP titrations was measured using an Oxygraph‐2k (O2k, Oroboros Instruments, Innsbruck, Austria). After air calibration, Blebbistatin was added to block the spontaneous fiber contraction. Malate (5 mM), glutamate (10 mM) and succinate (10 mM) were then added followed by 8 serial ADP titrations (ADP1 to ADP8), starting at 31.25 μM up to a final concentration of 2 mM. The DatLab 4 software (OROBOROS Instruments) was used to assess Oxygen flux levels. Then, the ‘bioenergetic units’ [ng atom O·min^−1^·mg^−1^ = ng atom O·min^−1^·mg^−1^ = lmol O·min^−1^·g^−1^] was converted by the multiplication factor [nmol O_2_·s^−1^·g^−1^ = pmol O_2_·s^−1^·mg^−1^]: 1000/(2·60) = 8.33 as previously described [16], to estimate flux in the International System of Units (SI), namely, pmol/(s*mg). Experiments when respiration increased more than 15% after the addition of Cytochrome C (10 μM) were discarded, as it indicates loss of integrity of the mitochondrial membrane. Then, the Submaximal State 3, corresponding to the 5^th^ ADP titration, was then used to represent mitochondrial respiration (Mit‐O_2_ flux).

### 2.3 Muscle Fiber‐ratio

Muscle samples from vastus lateralis biopsies were frozen at −150°C in liquid nitrogen cooled isopentane. Frozen samples were kept at −80°C until sectioning. Samples were equilibrated in the −25°C chamber of a Leica CM1950 Cryostat for at least 40 min, after which 10 μm sections were cut onto glass slides (Tissue Tack, Polysciences). Sections on slides were air‐dried at room temperature for 1 h before processing with immunofluorescence. Briefly, sections on slides were fixed with 3.7% formaldehyde (methanol‐free, Polysciences) for 10 min at room temperature (RT), then permeabilized with 0.2% Triton X‐100 (Sigma) for 10 min at RT, and then blocked with 10% normal goat serum (Sigma) for 1 h at 37°C. Sections were then stained overnight at 4°C with the following antibodies: for type 1 muscle fibers (slow) A4.840 hybridoma (Developmental Studies Hybridoma Bank, DSHB) at 1:1 concentration, while for type 2 muscle fibers (fast) A4.74 hybridoma (DSHB) at 1:4 concentration. After washing, appropriate Alexa Fluor conjugated secondary antibodies (Invitrogen) were used at 1:200 concentration for 40 min at 37°C. After washing, coverslips were mounted on the slides with Prolong Glass anti‐fade mounting media (Invitrogen). Slides were imaged with a Zeiss LSM 980 confocal microscope with a 20×, NA 0.7 objective. Z‐stacks of 3 × 3 tiles were obtained, and the maximum intensity projections of those tiles scored for slow (red fluorescence) or fast (green fluorescence) muscle fibers. As not enough muscle sample was available for microscopy, in 30 participants we estimated fiber‐ratio based on the ratio between myosin specific of type I or type II fibers from a previous muscle proteomic analysis [17]. Age and sex as independent variables significantly predicted microscopy estimated fiber‐ratio. This model was used to estimate fiber‐ratio in the sample with missing microscopy.

### 2.4 Muscle Mass Evaluation

Muscle mass was assessed using the D3‐creatine dilution technique as previously described [18, 19]. Participants ingested 30 mg of stable isotope‐labelled creatine (D3‐creatine) and after 72 h, they provide a fasting urine sample analysed via liquid chromatography and tandem mass spectrometry (MS/MS) to measures D3‐creatinine, unlabelled creatinine, and creatine levels. These values were imputed with an algorithm previously validated, using the percent of enrichment with spillage correction to calculate total body creatine pool size [20]. Notably, this method relies on the enrichment ratio of D3‐creatinine to unlabelled creatinine, not creatinine clearance or renal function. Since not all participants in the GESTALT study underwent the D3‐creatine dilution technique (with 46/82 measures available), we employed a predictive model using VO_2_, age and sex as features to estimate muscle mass for those lacking direct measurements. Supplementary Methods (S1) provides extendend information on muscle sample preparation, RNA‐sequencing and bioinformatic pipeline used in this study.

## 3 Results

### 3.1 GESTALT Features and Energetic Fitness Association With age, Muscle Mass and Muscle Fiber‐ratio

**FIGURE 1:**
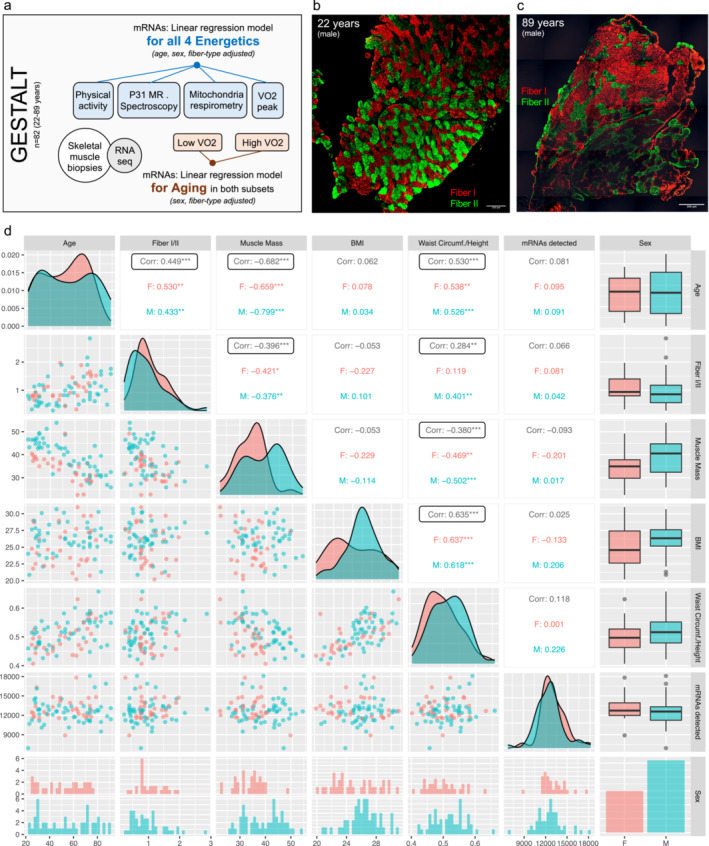
GESTALT study participants and skeletal muscle features. (a) Schematic representation of the study design. (b) Representative images of muscle fiber‐types from two GESTALT participants: a 22‐year‐old male (scale bar: 250 μm) and an 89‐year‐old male (scale bar: 100 μm), obtained through microscopy. (c) Cross‐correlation matrix displaying the relationships among various features of GESTALT participants, including age, fiber‐type I/II ratio, muscle mass, body mass index (BMI), waist circumference/height (central obesity index), number of mRNAs detected by Illumina RNA Sequencing, and sex. The colours represent the sex of the participants (light blue: men, pink: women). Pearson's correlation was used (*p ≤ 0.05, **p ≤ 0.01, ***p ≤ 0.001).

A summary of participants characteristics is shown in Supplementary Table S4. Cross‐correlations among participants and muscle features are displayed in Figure 1d, while here we report only the significant associations (*p* < 0.05). Even in this very healthy population the proportion of type I muscle fibers (slow) increased significantly with age (*r* = 0.44) [4, 21]. Figure 1b,c shows representative images from a younger (22 years old) and older (89 years old) GESTALT participant. Fiber‐ratio negatively correlated with muscle mass (*r* = −0.39) while positively with central obesity in males only. Also, muscle mass negatively correlated with age (*r* = −0.68). Waist‐circumference/height positively correlated with both age and BMI (*r* = 0.53 and 0.63 respectively). Correlation between the for the four energetic measures with age, muscle mass and muscle fiber type is reported in Supplementary Figure S5, while here we report only the significant findings. We observed an age‐dependent decline of all four energetic measures, which was statistically significant for VO_2_ (*r* ^2^ = 0.39) and kPCr (*r* ^2^ = 0.13). Muscle mass was strongly associated with VO_2_ (*r* ^2^ = 0.53) and kPCr (*r* ^2^ = 0.05). VO_2_ was positively associated with PA (*r* ^2^ = 0.04), and Fiber‐ratio (*r* ^2^ = 0.06). kPCr was positively associated with both PA (*r* ^2^ = 0.21) and VO_2_ (*r* ^2^ = 0.19). Mit‐O_2_ flux was positively but not significantly associated with any of the other energetic measures.

### 3.2 Differential Gene Expression (DGE) Profiles Across Energetic Fitness Measures

**FIGURE 2:**
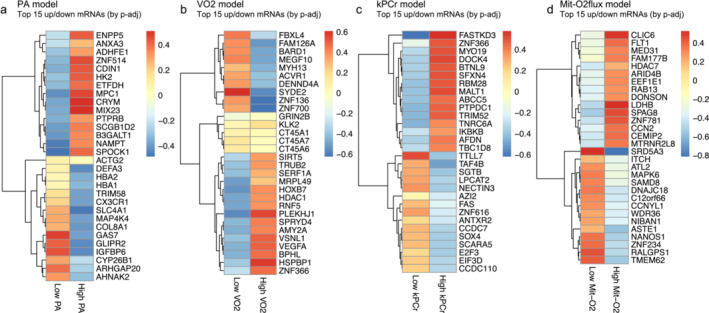
Differential gene expression (DGE) analysis for the four energetic fitness measurements. Heatmaps displaying the top 15 up‐regulated and top 15 down‐regulated protein‐coding mRNA genes (*p* < 0.01) that are differentially expressed according to the respective energetic variables. (a) PA, (b) VO_2_, (c) kPCr, (d) MitO_2_flux. The distribution of each energetic measure was divided into tertiles, and the first (lowest) and third (highest) tertiles are visualized. Red indicates up‐regulated genes, while blue indicates down‐regulated genes.

Differential gene expression (DGE) analysis was conducted using linear regression models for all four different energetic measures, adjusting for age, muscle fiber‐ratio, sex and batch effect (Figure 1a). The top 15 over‐ and under‐expressed protein‐coding genes are shown as heatmap plot in Figure 2 for all four energetic measures. Information on mRNA transcripts detected in DGE are available in Supplementary Data S6 (sheets 1–4, see look‐up table in Supplementary Data S7). Unique mRNA transcripts are available in Supplementary Data S6 (sheets 5–6). Top five most significant protein‐coding genes for PA (50 up‐ and 205 down‐regulated), VO_2_ (75 up‐ and 72 down‐regulated), kPCr (45 up‐ and 32 down‐regulated) and Mit‐O_2_ flux (60 up‐ and 41 down‐regulated) are listed in Supplementary Table S8, including muscle function and references. Significant, differentially expressed mitochondria‐ and splicing‐related genes [22, 23] are labelled in volcano plot for each model in Supplementary Figure S9, with top 3 mitochondria‐related genes in Supplementary Table S10 and splicing‐related genes in Supplementary Table S11. Genes significantly up‐ and down‐regulated in at least two energetic measures are listed in Supplementary Table S12.

### 3.3 Mitochondrial Physiology and Respiration‐Related Pathways Are Shared Across Energetic Measures Examined

**FIGURE 3:**
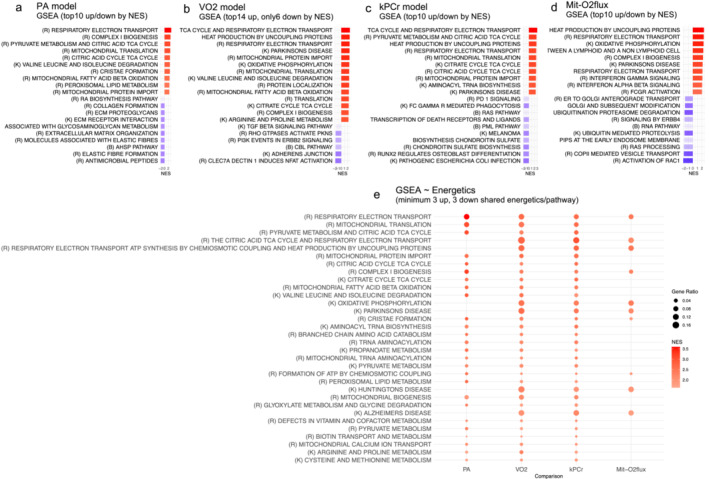
Gene Set Enrichment Analysis (GSEA) results for the four energetic fitness measurements. (a) PA (physical activity) model, (b) VO_2_ model, (c) kPCr model, (d) Mit‐O2 flux model. In panels (a), (c), and (d), the top 10 significantly up‐regulated pathways (red) and top 10 significantly down‐regulated pathways (blue) are shown (*p*‐adjusted < 0.05). For panel (b), as there were fewer than six down‐regulated pathways, the top 14 up‐regulated pathways are displayed. The gradient from blue to red corresponds to the normalized enrichment score (NES), and the colour intensity indicates the level of significance (*p*‐adjusted < 0.05 for all pathways shown, more information in Supplementary S6 (sheets 7‐10)). (e) Pathways that are significantly enriched (*p*‐adjusted < 0.05) in at least three of the four energetic measurements are shown. The size of the dot represents the Gene Ratio, while the gradient from blue to red is mapped to the normalized enrichment score (NES).

Gene Set Enrichment Analysis (GSEA) was used to estimate biological processes associated with energetic fitness and exercise. The energetic measure associated with the highest number of significant pathways was PA with 223 (156 down‐ and 67 up‐regulated), followed by VO_2_ with 158 (6 down‐ and 152 up‐regulated), kPCr with 122 (71 down‐ and 51 up‐regulated) and lastly Mit‐O_2_ flux with 47 (13 down‐ and 34 up‐regulated) (Supplementary S6; sheets 7–10). Figure 3a–d shows top 10 down‐ and up‐regulated pathways in all four models). Shared pathways across energetic measures illustrated in Figure 3e were primarily associated with mitochondria‐related processes. No shared down‐regulated pathways in at least three energetic measures were found.

We examined pathways enriched for only one of the four energetic variables (Supplementary S6, sheets 11–14). The number of unique pathways detected was as follows: 130 for PA (107 down‐ and 23 up‐regulated), 84 for VO_2_ (6 down‐ and 78 up‐regulated), 31 for kPCr (27 down‐ and 4 up‐regulated) and 18 for Mit‐O_2_ flux (12 down‐ and 6 up‐regulated). Physical activity top 10 unique up‐regulated pathways were mainly related to translation (initiation, synthesis and elongation), DNA repair and RNA degradation. For VO_2_ model, top unique pathways were associated with to amino acid metabolism and biological oxidations. The kPCr model targeted splicing processing and transcription, consistent with the notion that adaptation to different level of energetics is associated with the production of modulation of alternative splicing. Lastly, the unique gene sets in the mitochondrial respirometry pointed to immunity and inflammation. Interpreting the unique down‐regulated pathways was challenging because of substantial inconsistency between models. In the PA model, the top 10 unique down‐regulated pathways were primarily related to the extracellular matrix (ECM), its components such as collagen and glycoproteins, interactions with cell surface receptors like integrins, and processes like ECM assembly, remodelling and degradation. The VO_2_ model had only six down‐regulated pathways, mainly involving immune response, and cytoskeletal organization. The kPCr model showed down‐regulation of various cellular processes and signalling events, including development, cancer, signalling cascades and structural components of cells. Lastly, the unique down‐regulated gene sets in the Mit‐O_2_ flux model pointed to a mixed variety of cellular processes.

### 3.4 Energetics‐Linked Alternative Splicing Variants Are Enriched for Pathways Involving Cytoplasmic Ribonucleoprotein Granules

**FIGURE 4:**
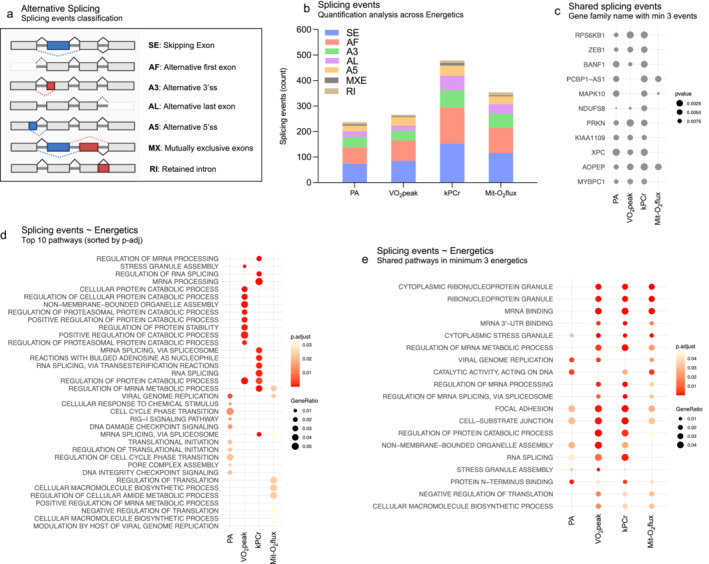
Alternative splicing (AS) analysis. (a) Classification of different types of alternative splicing events. (b) Characterization of alternative splicing events for each energetic model, showing the distribution of event types. (c) Gene names that are shared across alternative splicing events in at least three of the four energetic models. (d) Top 10 biological pathways identified by over‐representation analysis (ORA) based on gene family names from the alternative splicing events. (e) Pathways (ORA) that are significantly enriched (*p*‐adjusted < 0.05) in alternative splicing events from at least three of the four energetic models. The gradient colour represents the adjusted *p*‐value, while the size of the dots corresponds to the gene ratio.

Previous research suggested that muscles adapt to different level of energetics and physical activity by producing different splicing variants, which have been fully characterized [5, 17]. We identified alternative splicing (AS) events that were differentially expressed according to different measures of energetics, see Figure 4a. Across all energetic measures, exon skipping (SE) was the most common AS event, followed by alternative first exon (AF), alternative 3′ splice site or acceptor site (A3), alternative last exon (AL), alternative 5′ splice site or donor site (A5), mutually exclusive exons (MX/MXE), and intron retention (RI), as depicted on event counts per energetic measure in Figure 4b. Overall, the model with the highest number of significant splicing events (*p* < 0.01) was kPCr (479 events), followed by Mit‐O2 flux (350 events), VO_2_ (266 events) and PA (237 events). Detailed information on significant splicing events for each energetic measure is available in (Supplementary S6 sheets 15–18). Figure 4c shows the gene family names that are shared across alternative splicing events in at least three of the four energetic models, while Supplementary Table S13 describe their function and provide references.

To gain insights into the function of the identified splicing events, we conducted enrichment analyses using Over‐Representation Analysis (ORA). Alternative splicing events were enriched (*p*‐adjusted < 0.05) were: 64 for PA, 104 for VO_2_, 88 for kPCr and 58 for Mit‐O_2_ flux model (Supplementary S6, sheets 19–22). The direct MRS measure of ‘in vivo’ of mitochondrial function (kPCr) produced spliced transcripts variants enriched for ‘alternative splicing’, confirming the notion that alternative splicing regulates itself recursively. Top 10 most significant pathways enriched by splicing event as an energetic function are shown in Figure 4.

Remarkably, among splicing pathways enriched for at least three energetic measures (Figure 4e) many of the significant enriched pathways pointed to the formation of cytoplasmic ribonucleoprotein granules, as well as mRNA binding, mRNA UTR binding, stress granule assembly and the regulation of mRNA splicing, strongly suggesting that they play an important role in muscle adaptation to different levels of fitness. Cytoplasmic ribonucleoprotein granules are thought to be a stress response mechanism [24, 25] but their physiological role in adult skeletal muscle and adaptation to exercise is currently unknown. RNA binding proteins are involved in muscle adaptation in response to repeated contractile activity and it has been suggested that they exert this activity by recognizing specific sequences in RNAs binding sites, including UTRs [26].

### 3.5 Aging in Low VO_2_ Cohort Exhibits Enhanced Splicing Processes and Increased Splicing Events Consistent With the Energy‐Splicing Axis Hypothesis of Aging

We had previously hypothesized that the production of splicing variants is a resilience mechanism activated in skeletal muscle when energy becomes scarce. To address this hypothesis, we compared GESTALT participants in the lower and higher tertile of VO_2_. We then conducted parallel analyses on the first (low) and third (high) cohort using two distinct models adjusted for sex and batch effects.

**FIGURE 5:**
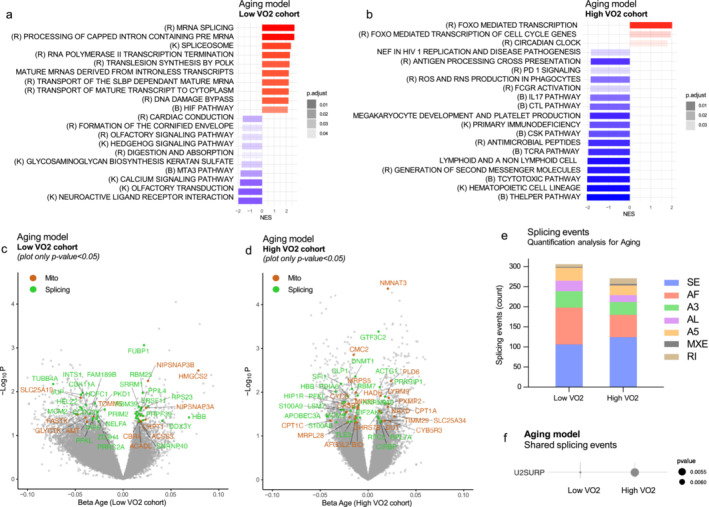
Aging model in low and high cardiovascular fitness (VO_2_) cohorts. (a) Low VO_2_ cohort and (b) high VO_2_ cohort: Gene Set Enrichment Analysis (GSEA) results for aging model. Top 10 significantly up‐regulated pathways (red) and top 10 significantly down‐regulated pathways (blue) are shown (*p*‐adjusted < 0.05). For panel (b), since there were only three up‐regulated pathways, the top 17 underregulated pathways are displayed. The gradient from blue to red corresponds to the normalized enrichment score (NES), and the colour intensity indicates the level of significance (*p*‐adjusted < 0.05 for all pathways). (c) Low VO_2_ cohort and (d) High VO_2_ cohort: Volcano plots for low and high VO_2_ cohort models for aging, respectively. Significant mitochondrial‐related genes [22] are shown in brown, and splicing‐related genes [23] are shown in green. The *x*‐axis represents the beta coefficient estimated from linear regressions, and the *y*‐axis represents the negative log of the *p*‐value. Due to stringent significance cut‐offs, only for visualization purposes genes with *p* < 0.05 were labelled. (e) Characterization of alternative splicing events in both cohorts, showing the distribution of event types. (f) Gene names that are shared across alternative splicing events in both cohorts.

Aging model in participants with lower VO_2_ identified 17 up‐ and 28 down‐regulated significant protein‐coding mRNAs (Supplementary S6, sheet 23), while in participants with higher VO_2_ we detected 66 up‐ and 96 significant down‐regulated protein‐coding mRNAs (*p* < 0.01, Supplementary S6, sheet 24). In the higher VO_2_ cohort only 3 GSEA pathways were up‐regulated with aging (Figure 5b, Supplementary S6, sheet 26), including circadian rhythm and *FOXO*‐mediated transcription; the latter is an intriguing finding, as it was found to promote longevity through beneficial effect on stress resistance, metabolism and proteostasis [27, 28]. Down‐regulated pathways were related to immune system network, confirming that high physical activity offsets the effect of aging on inflammation. In the lower VO_2_ group, the top 3 most up‐regulated pathways were splicing‐related, suggesting splicing machinery plays a key role in aging for sedentary individuals (Supplementary S6, sheet 25 and Figure 5a). Additionally, pathways related to DNA damage and hypoxia inducible factors (HIFs) were also up‐regulated.

Figure 5c visually depicts the positive enrichment of splicing‐related mRNAs in the lower VO_2_ group. In contrast, the higher VO_2_ group shows a more heterogeneous enrichment proportion of mitochondrial and splicing‐related genes (Figure 5d). The count of splicing events was generally higher in the low VO_2_ aging model compared with the high VO_2_ cohort (306 vs. 271, respectively, Supplementary S6, sheets 27–28 and Figure 5e). [29]. Only the mRNA coding for the spliceosome factor *U2SURP* was characterized by having a splicing event in both VO_2_ cohorts (Figure 5f).

In the lower VO_2_ group, enriched pathways involved cellular catabolism, cell cycle, RNA localization and nuclear transport regulation (Supplementary Figure S14a). Conversely, those with higher VO_2_ exhibited enrichment in pathways enhancing splicing, chromosome organization, ubiquitination and viral–host interactions. The overlapping pathways found in the top 100 results of enrichment analysis for both low and high VO_2_ cohort are depicted in Supplementary Figure S14b. These findings suggest that during the aging process, splicing events in sedentary individuals impact many important cellular processes, such as cellular breakdown and cell division that remain unaffected in those who are physically active.

## 4 Discussion

This study examined the association between muscle transcriptomics and four distinct domains of energetics in muscle biopsies collected from 50 men and 32 women who were extremely healthy according to rigid clinical and laboratory criteria.

We confirmed previous literature detecting progressive reduction of muscle fiber‐type II, suggesting that these fibers are lost with aging at an accelerated rate even in healthy individuals. All the energetic measures declined with aging and in general were positively correlated, although they capture substantially different aspects of energetics. A puzzling result was that mitochondrial respiration did not exhibit significant correlations with the other energy measures. This finding contrasts with a previous study conducted in a relatively smaller sample size (*n* = 18) of younger individuals where kPCr and Mit‐O_2_ flux were found positively correlated [30]. A possible explanation is that more global measure of fitness and energetic are affected by age‐related differences of cardiovascular function and muscle perfusion that do not affect Mit‐O_2_ flux [31, 32].

Transcriptomic analyses revealed model‐specific differentially expressed mRNAs. Nonetheless, pathway enrichment analyses consistently identified oxidative phosphorylation and mitochondrial homeostasis across models. This discrepancy likely arises from statistical variability in individual transcript *p*‐value rankings, underscoring the robustness of pathway‐level insights. Enrichment analysis, and particularly GSEA, allowed us to identify subtle yet coordinated changes in complex gene expression datasets, offering a holistic view of gene expression data that may be missed by single‐gene analyses. By examining the gene sets enriched by significant splicing events in ORA, we consistently found evidence of amplification of the action of the spliceosome and mRNA binding protein activity, both of which have been previously implicated in muscle adaptation to different levels of physical activity [26]. We found evidence of enhanced formation of cytoplasmic ribonucleoprotein (RNP) granules and their assembly in association with mRNA binding and regulation in all four models. The first study demonstrating the relationship between ribonucleoproteins and myofibrils physiology was published over 70 years ago [33]. More recently, two studies have revealed the connection between mRNP granules and muscle stem cells [34] and the formation of amyloid‐like myo‐granules in regenerating muscle driven by TDP‐43 [35]. More research is needed to clarify this relationship.

In this study, we also investigated the splicing changes in cross‐sectional aging from a subset of low and high cardiovascular fitness GESTALT participants. Interestingly, only in the lower fitness (VO_2_) cohort, the main three overexpressed pathways across aging were splicing‐related, confirming evidence of the energy‐splicing axis hypothesis of aging which proposed an inverse association between mitochondrial and energy status with RNA splicing, acting as a resilience strategy to preserve energetic homeostasis in aging [6]. The splicing events identified in the low fitness cohort enriched pathways related to cellular homeostasis regulation, supporting the hypothesis that these splicing variants may act to counteract cellular dysregulation, which is frequently associated with various age‐related diseases.

In general, a comprehensive interpretation of the role of differentially expressed splicing variants might be complex because the biological function of many of these variants remains unknown. Studies that have attempted to validate the effects of splicing variants by targeted proteomics have been challenging and only partially successful. Developing a transcriptomic‐proteomic pipeline for the validation and study of splicing variants is an important goal of future research. As splicing was shown to be affected by transposon‐mediated interference and DNA methylation at acceptor and donor splice sites [36], future studies should investigate the relationship between transposon positions and the emergence of splicing variants.

Our study has limitation. First, due to the cross‐sectional nature of the study, it cannot establish direct causal pathways between gene expression changes and the assessed energetic variables. Future research should selectively evaluate the impact of interventions aimed at enhancing mitochondrial function, independent of physical activity. Second, this healthy aging population may not be the most suitable design for understanding biological responses to ‘low energy availability’. Future studies should include participants affected by relevant pathology and frailty to investigate whether mechanisms of muscle adaptation to fitness are different. Third, the self‐reported nature of the physical activity measure may have introduced substantial measurement error, therefore future studies should replicate these findings using objective measures (i.e. wearable accelerometers). Fourth, the short‐read Illumina platform used for reconstructing splicing variants, struggles to detect certain splicing variants, especially long or novel isoforms [37]. Employing a hybrid approach of short‐read and long‐read sequencing may offer increased sensitivity and specificity in identifying splicing variants [38, 39, 40].

In conclusion, have identified distinctive splicing isoforms specific to each energetic measurement and analysed their enrichment at the pathway level. We confirm the major involvement of mitochondria‐related mRNAs in directly shaping multiple energetic aspects related to improved muscular respiration and overall wide‐ranging muscle health. We provide evidence that the energy‐splicing axis hypothesis of aging operates in conditions of lower energetic fitness to maintain healthy cellular homeostasis regulation. This analysis sheds light on the biological mechanisms connecting muscle health and mitochondria, encompassing both shared and distinct elements among the four energetic models investigated.

## Conflicts of Interest

The authors declare no conflicts of interest.

## Supporting information

Supplementary materials.

## Data Availability

Access to GESTALT data is available upon review and subsequent approval of proposals submitted through the BLSA study website (https://www.blsa.nih.gov/). The R script used can be found at https://github.com/niairpnih/energetics2024.
