# Supplementary material for: Skeletal Muscle mRNA Splicing Variants Association With Four Different Fitness and Energetic Measures in the GESTALT Study
Source: J Cachexia Sarcopenia Muscle. 2024 Dec 2;16(1):e13603. doi: 10.1002/jcsm.13603 (PMC11695105; doi:10.1002/jcsm.13603)
Supplement: Supplementary file 1 — Supplementary materials. [file JCSM-16-e13603-s001.zip › S1_Supplementary S1_Methods.pdf]

S1

Supplementary Methods

1. Muscle sample preparation and RNA-sequencing

Total RNA from muscle biopsies was extracted using Qiagen Fibrous Tissue kit (Qiagen) and cDNA was synthesized using the NuGen Ovation RNA-seq system v2. Libraries were generated with the TruSeq ChIP Library Preparation Kit (Sets A [IP-202-1012] and B [IP-202-1024]). RNA was sequenced using the Illumina HiSeq 2500 sequencing system with average depth of 148 million single-end reads and ranging between 66 million and 299 million reads. The read-length was on average 134 bases. The mean mapping rate calculated from the logs generated by the STAR aligner was 84.93%. Furthermore, 7.7% of reads mapped to multiple loci, indicating that more than 90% of total reads were mapped to the reference genome.

2. Bioinformatic pipeline and statistical analysis

After initially assessing the quality of reads using FastQC v. 0.11.8, the tool BBDuk v. 38.73, which is part of the BBTools package (http://sourceforge.net/projects/bbmap/), was used to trim adapter sequences. Next, FastQC was rerun to assess the quality of cleaned fastq files. Subsequently, reads were aligned to the hg38 reference genome along with ENSEMBL annotation v. 104, using STAR v. 2.7.8.a in two-pass mode. Next, the generated BAM files were sorted and indexed using Samtools version 1.9. FeatureCounts, which requires BAM files, was used to quantify expression at the gene level, and Kallisto v. 0.48.0 which requires fastq files, was run in bootstrap mode (-b 100) to quantify expression at the transcript level. Finally, an overall assessment of the outputs of the bioinformatics pipeline was conducted using MultiQC v. 1.14. Out of the 60,605 RNAs in the Ensembl hg38 v104 database, we identified 26,891 RNAs (26,711 RNAs for Mit-O_2 flux model) that had more than 5 aligned reads in at least half of the samples. The RNA-alignment QC metrics (after running STAR) are detailed in Supplementary Table (S2).

To perform Differential Gene Expression (DGE) analysis, DESeq2 v. 1.36.0 was used for sample normalization as well as for DGE analysis. Statistical significance was defined as p-value (p) <0.01. To detect and quantify alternative splicing forms, SUPPA2 was used to first generate an "ioe" file using the generateEvents parameter, and subsequently a PSI (Percentage Spliced In) matrix was generated from the transcript expression matrix using the psiPerEvent parameter. The PSI matrix generated by Suppa2 was filtered by requiring all samples for an event to have PSI values > 0.10. Following this step, the filtered PSI matrix was used as input to a linear model. Energetic model regression analyses were adjusted for age, gender, muscle fiber-ratio and sampling batch, while aging models were adjusted without the age confounder. ClusterProfiler 4.4.4 was used for Gene Set Enrichment Analysis (GSEA) and for Over Representation Analysis (ORA). For GSEA, we combined KEGG, BIOCARTA and REACTOME from the

Human Molecular Signatures Database (MSigDB). While GSEA uses the full dataset as input, ORA uses only the significant subset of genes identified though differential gene expression analysis. ORA for Alternative Splicing (AS) using Gene Ontology (GO) default dataset, was performed using a subset of the output of the linear model using a threshold of $p < 0.1$, and detected genes (26,711 RNAs for Mit-O_2 flux, and 26,891 for other analysis) as background. We considered as significant only those ontologies with p-adjusted (p-adj) <0.05. Following this step, the filtered PSI matrix was used as input to a linear model. All regression analyses were controlled for age, muscle fiber-ratio, sex and sampling batch. Figures were created using ggplot2 package in R, Microsoft Office suite (version 16.81) and GraphPad Prism (version 10.1.1). Methods and bioinformatic software are listed in Supplementary Table (S3).
