# Supplementary material for: Skeletal Muscle mRNA Splicing Variants Association With Four Different Fitness and Energetic Measures in the GESTALT Study
Source: J Cachexia Sarcopenia Muscle. 2024 Dec 2;16(1):e13603. doi: 10.1002/jcsm.13603 (PMC11695105; doi:10.1002/jcsm.13603)
Supplement: Supplementary file 1 — Supplementary materials. [file JCSM-16-e13603-s001.zip › S2_Supplementary Table S2.pdf]

S2

	Mean	Median	Minimum	Maximum	Standard Deviation
**Raw Reads**	148,110,494.01	136,664,093.50	66,042,735.00	299,402,360.00	56,463,085.69
**Read Length**	134.01	135	114	138	3.5
**Unique Reads**					
**Unique Reads**	125,322,453.73	115,539,728.00	54,698,394.00	253,613,385.00	46,528,363.99
**Unique Reads (%)**	84.93	84.25	73.74	93.43	4.09
**Mismatches**					
**Mismatch Rate (%)**	0.38	0.36	0.23	0.72	0.09
**Deletion Rate (%)**	0.01	0.01	0.01	0.02	0
**Deletion Length**	1.45	1.38	1.18	1.87	0.19
**Insertion Rate (%)**	0.01	0.01	0	0.02	0.01
**Insertion Length**	1.87	1.78	1.32	3.17	0.29
**Multi-Mapping Reads**					
**Mapped to Multiple Loci**	11,984,162.35	9,525,057.00	2,875,994.00	27,559,234.00	6,920,967.57
**Mapped to Multiple Loci (%)**	7.65	7.72	3.8	11.96	2.12
**Mapped to too many loci**	477,114.40	249,512.50	29,320.00	3,215,837.00	582,010.48
**Mapped to too many loci (%)**	0.3	0.17	0.03	1.32	0.32
**Unmapped Reads**					
**Unmapped: Too Short**	8,825,219.11	8,483,072.00	2,326,592.00	21,858,082.00	4,060,006.61
**Unmapped Too Short (%)**	6.16	5.77	2.04	12.51	2.42
**Reads Unmapped: Other (%)**	0.96	0.6	0.12	7.72	1.05

Table S2: Post-alignment (STAR) QC metrics for 82 samples sequenced in this study
