# Supplementary material for: Skeletal Muscle mRNA Splicing Variants Association With Four Different Fitness and Energetic Measures in the GESTALT Study
Source: J Cachexia Sarcopenia Muscle. 2024 Dec 2;16(1):e13603. doi: 10.1002/jcsm.13603 (PMC11695105; doi:10.1002/jcsm.13603)
Supplement: Supplementary file 1 — Supplementary materials. [file JCSM-16-e13603-s001.zip › S3_Supplementary Table S3.pdf]

S3

Material and Method		Reference
**Methods**	Immunofluorescence staining	[1]
**Bioinformatic software**	FastQC v. 0.11.8	[2]
	STAR v. 2.7.8.a	[3]
	Samtools version 1.9	[4]
	FeatureCounts	[5]
	Kallisto v. 0.48.0	[6]
	MultiQC v. 1.14	[7]
	DESeq2 v. 1.36.0	[8]
	SUPPA2	[9]
	ClusterProfiler 4.4.4	[10]

Table S3: Methods and Software used for bioinformatic analysis with relative references.

1. Partridge, J.J., et al., *DNA damage modulates nucleolar interaction of the Werner protein with the AAA ATPase p97/VCP.* Mol Biol Cell, 2003. **14**(10): p. 4221-9.
2. Andrews, S., *FastQC: A Quality Control Tool for High Throughput Sequence Data.* 2010.
3. Dobin, A., et al., *STAR: ultrafast universal RNA-seq aligner.* Bioinformatics, 2013. **29**(1): p. 15-21.
4. Li, H., et al., *The Sequence Alignment/Map format and SAMtools.* Bioinformatics, 2009. **25**(16): p. 2078-9.
5. Liao, Y., G.K. Smyth, and W. Shi, *featureCounts: an efficient general purpose program for assigning sequence reads to genomic features.* Bioinformatics, 2014. **30**(7): p. 923-30.
6. Bray, N.L., et al., *Near-optimal probabilistic RNA-seq quantification.* Nat Biotechnol, 2016. **34**(5): p. 525-7.
7. Ewels, P., et al., *MultiQC: summarize analysis results for multiple tools and samples in a single report.* Bioinformatics, 2016. **32**(19): p. 3047-8.
8. Love, M.I., W. Huber, and S. Anders, *Moderated estimation of fold change and dispersion for RNA-seq data with DESeq2.* Genome Biol, 2014. **15**(12): p. 550.
9. Trincado, J.L., et al., *SUPPA2: fast, accurate, and uncertainty-aware differential splicing analysis across multiple conditions.* Genome Biol, 2018. **19**(1): p. 40.
10. Yu, G., et al., *clusterProfiler: an R package for comparing biological themes among gene clusters.* OMICS, 2012. **16**(5): p. 284-7.
