# Supplementary material for: Skeletal Muscle mRNA Splicing Variants Association With Four Different Fitness and Energetic Measures in the GESTALT Study
Source: J Cachexia Sarcopenia Muscle. 2024 Dec 2;16(1):e13603. doi: 10.1002/jcsm.13603 (PMC11695105; doi:10.1002/jcsm.13603)
Supplement: Supplementary file 1 — Supplementary materials. [file JCSM-16-e13603-s001.zip › S4_Supplementary Table S4.pdf]

S4

Parameters		Mean ± SD (or %)
**Age**	Years	53.17 ± 19.51
**Gender**	Men	52 (63.41 %)
	Women	30 (36.59 %)
**Race**	White	68 (82.92 %)
	Black	11 (13.41 %)
	Asian	3 (3.65 %)
**Physical parameters**	BMI (kg/m^2)	25.83 ± 2.57
	Waist Circumference/Height	0.511 ± 0.051

Table S4: GESTALT participants characteristics (n=82)
