# Supplementary material for: Skeletal Muscle mRNA Splicing Variants Association With Four Different Fitness and Energetic Measures in the GESTALT Study
Source: J Cachexia Sarcopenia Muscle. 2024 Dec 2;16(1):e13603. doi: 10.1002/jcsm.13603 (PMC11695105; doi:10.1002/jcsm.13603)
Supplement: Supplementary file 1 — Supplementary materials. [file JCSM-16-e13603-s001.zip › S7_Supplementary S7_Data (look-up table for S6).pdf]

S7

Supplementary Data (look-up table for S6)

Sheet 1_DGE PA: Differential Gene Expression (DGE) analysis for physical activity (PA) model. Gene row name, base mean, log2 fold change (ß), lfc-SE (ß-SE), stat, p-val, p-adj, gene description, chromosome scaffold name, gene start bp, gene end bp, strand, karyotype band, gene name, gene GC content, gene type (biotype), miRbase transcript name ID (if available), and gene length are provided

Sheet2_DGE VO2: Differential Gene Expression (DGE) analysis for VO_2 peak model. Gene row name, base mean, log2 fold change (ß), lfc-SE (ß-SE), stat, p-val, p-adj, gene description, chromosome scaffold name, gene start bp, gene end bp, strand, karyotype band, gene name, gene GC content, gene type (biotype), miRbase transcript name ID (if available), and gene length are provided

Sheet 3_DGE kPCr: Differential Gene Expression (DGE) analysis for kPCr model. Gene row name, base mean, log2 fold change (ß), lfc-SE (ß-SE), stat, p-val, p-adj, gene description, chromosome scaffold name, gene start bp, gene end bp, strand, karyotype band, gene name, gene GC content, gene type (biotype), miRbase transcript name ID (if available), and gene length are provided

Sheet 4_DGE MitO2flux: Differential Gene Expression (DGE) analysis for Mit-O_2 flux model. Gene row name, base mean, log2 fold change (ß), lfc-SE (ß-SE), stat, p-val, p-adj, gene description, chromosome scaffold nname, gene start bp, gene end bp, strand, karyotype band, gene name, gene GC content, gene type (biotype), miRbase transcript name ID (if available), and gene length are provided

Sheet 5_DGE unique down: Unique down-regulated genes from DGE analysis for each of the four energetic measurements performed (filtered p-val<0.01). Gene stable ID, gene description, chromosome scaffold name, gene start bp, gene end bp, strand, karyotype band, gene name, gene GC content, gene type (biotype), miRbase transcript name ID (if available), and gene length are provided

Sheet 6_DGE unique up: Unique up-regulated genes from DGE analysis for each of the four energetic measurements performed (filtered p-val<0.01). Gene stable ID, gene description, chromosome scaffold name, gene start bp, gene end bp, strand, karyotype band, gene name, gene GC content, gene type (biotype), miRbase transcript name ID (if available), and gene length are provided

Sheet 7_GSEA PA: Gene Set Enrichment Analysis (GSEA) for physical activity (PA) model. Pathway, setSize, enrichment score, normalized enrichment score (NES), p-value, p-adjusted, q-value, rank, leading edge, core enrichment are provided

Sheet 8_GSEA VO2: Gene Set Enrichment Analysis (GSEA) for VO_2 peak model. Pathway, setSize, enrichment score, normalized enrichment score (NES), p-value, p-adjusted, q-value, rank, leading edge, core enrichment are provided

Sheet 9_GSEA kPCr: Gene Set Enrichment Analysis (GSEA) for kPCr model. Pathway, setSize, enrichment score, normalized enrichment score (NES), p-value, p-adjusted, q-value, rank, leading edge, core enrichment are provided

Sheet 10_GSEA MitO2flux: Gene Set Enrichment Analysis (GSEA) for Mit-O_2 flux model. Pathway, setSize, enrichment score, normalized enrichment score (NES), p-value, p-adjusted, q-value, rank, leading edge, core enrichment are provided

Sheet 11_GSEA PA_unique: Unique pathways from GSEA for PA measurement performed (filtered p-adj<0.05). Pathway, NES, p-adj, q-val, rank, leading edge, core enrichment for each model are provided

Sheet 12_GSEA VO2_unique: Unique pathways from GSEA for VO2 measurement performed (filtered p-adj<0.05). Pathway, NES, p-adj, q-val, rank, leading edge, core enrichment for each model are provided

Sheet 13_GSEA kPCr_unique: Unique pathways from GSEA for kPCr measurement performed (filtered p-adj<0.05). Pathway, NES, p-adj, q-val, rank, leading edge, core enrichment for each model are provided

Sheet 14_GSEA Mit-O2flux_unique: Unique pathways from GSEA for MitO2flux measurement performed (filtered p-adj<0.05). Pathway, NES, p-adj, q-val, rank, leading edge, core enrichment for each model are provided

Sheet 15_AS PA: Alternative splicing analysis (SUPPA2 software) for physical activity (PA) model. Gene row name, p-value, ß, AS event, gene description, chromosome scaffold name, gene start bp, gene end bp, strand, karyotype band, gene name, gene GC content, gene type (biotype), miRbase transcript name ID (if available), and gene length are provided

Sheet 16_AS VO2: Alternative splicing analysis (SUPPA2 software) for VO_2 peak model. Gene row name, p-value, ß, AS event, gene description, chromosome scaffold name, gene start bp, gene end bp, strand,

karyotype band, gene name, gene GC content, gene type (biotype), miRbase transcript name ID (if available), and gene length are provided

Sheet 17_AS kPCr: Alternative splicing analysis (SUPPA2 software) for kPCr model. Gene row name, p-value, ß, AS event, gene description, chromosome scaffold name, gene start bp, gene end bp, strand, karyotype band, gene name, gene GC content, gene type (biotype), miRbase transcript name ID (if available), and gene length are provided

Sheet 18_AS MitO2flux: Alternative splicing analysis (SUPPA2 software) for Mit-O_2 flux model. Gene row name, p-value, ß, AS event, gene description, chromosome scaffold name, gene start bp, gene end bp, strand, karyotype band, gene name, gene GC content, gene type (biotype), miRbase transcript name ID (if available), and gene length are provided

Sheet 19_ORA PA: Over-representation analysis (ORA) for physical activity (PA) model. Ontology source, gene set ID, pathway description, Gene Ratio, Bg ratio, p-value, p-adj, q value, Gene ID and count are provided

Sheet 20_ORA VO2: Over-representation analysis (ORA) for VO_2 peak model. Ontology source, gene set ID, pathway description, Gene Ratio, Bg ratio, p-value, p-adj, q value, Gene ID and count are provided

Sheet 21_ORA kPCr: Over-representation analysis (ORA) for kPCr model. Ontology source, gene set ID, pathway description, Gene Ratio, Bg ratio, p-value, p-adj, q value, Gene ID and count are provided

Sheet 22_ORA MitO2flux: Over-representation analysis (ORA) for Mit-O_2 flux model. Ontology source, gene set ID, pathway description, Gene Ratio, Bg ratio, p-value, p-adj, q value , Gene ID and count are provided

Sheet 23_Aging (VO2 Low cohort)_DGE: Differential Gene Expression (DGE) analysis for Aging model in Low VO_2 cohort. Gene row name, base mean, log2 fold change (ß), lfc-SE (ß-SE), stat, p-val, p-adj, gene description, chromosome scaffold name, gene start bp, gene end bp, strand, karyotype band, gene name, gene GC content, gene type (biotype), miRbase transcript name ID (if available), and gene length are provided

Sheet 24_Aging (VO2 High cohort)_DGE: Differential Gene Expression (DGE) analysis for Aging model in High VO_2 cohort. Gene row name, base mean, log2 fold change (ß), lfc-SE (ß-SE), stat, p-val, p-adj, gene description, chromosome scaffold name, gene start bp, gene end bp, strand, karyotype band, gene

name, gene GC content, gene type (biotype), miRbase transcript name ID (if available), and gene length are provided

Sheet 25_Aging (VO2 Low cohort)_GSEA: Gene Set Enrichment Analysis (GSEA) for Aging model in Low VO_2 cohort. Pathway, setSize, enrichment score, normalized enrichment score (NES), p-value, p-adjusted, q-value, rank, leading edge, core enrichment are provided

Sheet 26_Aging (VO2 High cohort)_GSEA: Gene Set Enrichment Analysis (GSEA) for Aging model in High VO_2 cohort. Pathway, setSize, enrichment score, normalized enrichment score (NES), p-value, p-adjusted, q-value, rank, leading edge, core enrichment are provided

Sheet 27_Aging (VO2 Low cohort)_AS: Alternative splicing analysis (SUPPA2 software) for Aging model in Low VO_2 cohort. Gene row name, p-value, ß, AS event, gene description, chromosome scaffold name, gene start bp, gene end bp, strand, karyotype band, gene name, gene GC content, gene type (biotype), miRbase transcript name ID (if available), and gene length are provided

Sheet 28_Aging (VO2 High cohort)_AS: Alternative splicing analysis (SUPPA2 software) for Aging model in High VO_2 cohort. Gene row name, p-value, ß, AS event, gene description, chromosome scaffold name, gene start bp, gene end bp, strand, karyotype band, gene name, gene GC content, gene type (biotype), miRbase transcript name ID (if available), and gene length are provided

Sheet 29_Aging (VO2 Low cohort)_ORA: Over-representation analysis (ORA) for Aging model in Low VO_2 cohort. Ontology source, gene set ID, pathway description, Gene Ratio, Bg ratio, p-value, p-adj, q value, Gene ID and count are provided

Sheet 30_Aging (VO2 High cohort)_ORA: Over-representation analysis (ORA) for Aging model in High VO_2 cohort. Ontology source, gene set ID, pathway description, Gene Ratio, Bg ratio, p-value, p-adj, q value, Gene ID and count are provided
