# Supplementary material for: Skeletal Muscle mRNA Splicing Variants Association With Four Different Fitness and Energetic Measures in the GESTALT Study
Source: J Cachexia Sarcopenia Muscle. 2024 Dec 2;16(1):e13603. doi: 10.1002/jcsm.13603 (PMC11695105; doi:10.1002/jcsm.13603)
Supplement: Supplementary file 1 — Supplementary materials. [file JCSM-16-e13603-s001.zip › S8_Supplementary Table S8.pdf]

S8

Model	Beta	Gene	Function	Reference
**PA**	Up	*PTPRB*	branching morphogenesis	[1]
		MIX23	mitochondrial protein import	[2]
		ENPP5	nucleotide and phospholipid signaling	[3]
		MPC1	mitochondrial pyruvate carrier subunit	[4]
		SCGB1D2	lipophilin subfamily	[5]
	Down	*GAS7*	muscle fiber composition transition	[6]
		HBA1	transporting oxygen in the blood	[7]
		HBA2		
		TRIM58	E3 ubiquitin ligase (late erythropoiesis)	[8]
		DEFA3	immune response (vs microbial pathogens)	[9]
**VO2**	Up	*VEGFA*	muscle regeneration, angiogenesis	[10-12]
		BPHL	serine hydrolase enzyme	[13]
		AMY2A	carbohydrate digestion	[14]
		HOXB7	stem cell differentiation	[15]
		SPRYD4	tumor suppressor	[16]
	Down	*KLK2*	protease-activated receptor	[17]
		FAM126A	synthesis of PI_4P	[18]
		ZNF136	transcriptional regulation in smooth muscle	[19]
		CT45A1	epithelial-mesenchymal transition	[20, 21]
		SYDE2	GTPases process to a GDP-bound state	[22]
**kPCr**	Up	*TRIM52*	NF-κB stress, inflammation, pyroptosis	[23-25]
		FASTKD3	energy balance of mitochondria under stress	[26]
		PTPDC1	putative role in muscle strength	[27]
		BTNL9	protease hydrolysis, ubiquitination	[28]
		MYO19	muscle contraction, mito-cristae design	[29, 30]
	Down	*AZI2*	inhibit DNA methylation	[31]
		LPCAT2	fatty acid composition, phosphatidylcholine	[32]
		CCDC7	cell proliferation regulation	[33]
		ANTXR2	cell surface receptor for anthrax toxin	[34]
		SGTB	co-chaperone function, ATPase activity	[35]
**MitO2flux**	Up	*MTRNR2L8*	bioenergetic stress biomarker	[36]
		CCN2	muscle hypertrophy regulation	[37]
		LDHB	pyruvate and lactate interconversion	[38]
		RAB13	tumors involvement (unknown in muscle)	[39]
		MED31	RNA pol II-dependent genes regulation	[40]
	Down	*SRD5A3*	glycosylation	[41]
		CCNYL1	WNT signaling	[42]
		C12orf66	protein-protein interaction	[43]
		NIBAN1	apoptosis regulation under stress	[44]
		DNAJC18	suggested for germ cell maturation	[45]

Table S8: Top five significant ($p<0.01$) up- and down-regulated protein-coding mRNAs (genes) for each of the four energetic measurements performed. Model, beta, gene name, function and reference from literature is provided

1. Soady, K.J., et al., *The receptor protein tyrosine phosphatase PTPRB negatively regulates FGF2-dependent branching morphogenesis.* Development, 2017. **144**(20): p. 3777-3788.
2. Zoller, E., et al., *The intermembrane space protein Mix23 is a novel stress-induced mitochondrial import factor.* J Biol Chem, 2020. **295**(43): p. 14686-14697.
3. Borza, R., et al., *Structure and function of the ecto-nucleotide pyrophosphatase/phosphodiesterase (ENPP) family: Tidying up diversity.* J Biol Chem, 2022. **298**(2): p. 101526.
4. Le, X.H., C.P. Lee, and A.H. Millar, *The mitochondrial pyruvate carrier (MPC) complex mediates one of three pyruvate-supplying pathways that sustain Arabidopsis respiratory metabolism.* Plant Cell, 2021. **33**(8): p. 2776-2793.
5. Lehrer, R.I., et al., *Secretory lipophilins: a tale of two species.* Ann N Y Acad Sci, 2000. **923**: p. 59-67.
6. Huang, B.T., et al., *Gas7-deficient mouse reveals roles in motor function and muscle fiber composition during aging.* PLoS One, 2012. **7**(5): p. e37702.
7. Gaspersic, J., et al., *Erythrocytosis: genes and pathways involved in disease development.* Blood Transfus, 2021. **19**(6): p. 518-532.
8. Thom, C.S., et al., *Trim58 degrades Dynein and regulates terminal erythropoiesis.* Dev Cell, 2014. **30**(6): p. 688-700.
9. Son, G.H., et al., *Whole blood RNA sequencing reveals a differential transcriptomic profile associated with cervical insufficiency: a pilot study.* Reprod Biol Endocrinol, 2021. **19**(1): p. 32.
10. Germani, A., et al., *Vascular endothelial growth factor modulates skeletal myoblast function.* Am J Pathol, 2003. **163**(4): p. 1417-28.
11. Arsic, N., et al., *Vascular endothelial growth factor stimulates skeletal muscle regeneration in vivo.* Mol Ther, 2004. **10**(5): p. 844-54.
12. Deasy, B.M., et al., *Effect of VEGF on the regenerative capacity of muscle stem cells in dystrophic skeletal muscle.* Mol Ther, 2009. **17**(10): p. 1788-98.
13. Ren, P., et al., *Inhibition of BPHL inhibits proliferation in lung carcinoma cell lines.* Transl Lung Cancer Res, 2023. **12**(5): p. 1051-1061.
14. Groot, P.C., et al., *Human pancreatic amylase is encoded by two different genes.* Nucleic Acids Res, 1988. **16**(10): p. 4724.
15. Tsuboi, M., et al., *The transcription factor HOXB7 regulates ERK kinase activity and thereby stimulates the motility and invasiveness of pancreatic cancer cells.* J Biol Chem, 2017. **292**(43): p. 17681-17702.

16. Ma, Z., et al., *Identification of SPRYD4 as a tumour suppressor predicts prognosis and correlates with immune infiltration in cholangiocarcinoma.* BMC Cancer, 2023. **23**(1): p. 404.
17. Skala, W., et al., *Structure-function analyses of human kallikrein-related peptidase 2 establish the 99-loop as master regulator of activity.* J Biol Chem, 2014. **289**(49): p. 34267-83.
18. Li, Y., et al., *FAM126A interacted with ENO1 mediates proliferation and metastasis in pancreatic cancer via PI3K/AKT signaling pathway.* Cell Death Discov, 2022. **8**(1): p. 248.
19. Vissing, H., et al., *Repression of transcriptional activity by heterologous KRAB domains present in zinc finger proteins.* FEBS Lett, 1995. **369**(2-3): p. 153-7.
20. Wen, M., et al., *CT45A1 promotes the metastasis of osteosarcoma cells in vitro and in vivo through beta-catenin.* Cell Death Dis, 2021. **12**(7): p. 650.
21. Shang, B., et al., *CT45A1 acts as a new proto-oncogene to trigger tumorigenesis and cancer metastasis.* Cell Death Dis, 2014. **5**(6): p. e1285.
22. Kouchi, Z. and M. Kojima, *Function of SYDE C2-RhoGAP family as signaling hubs for neuronal development deduced by computational analysis.* Sci Rep, 2022. **12**(1): p. 4325.
23. Liu, J., et al., *Tumor suppressor p53 cross-talks with TRIM family proteins.* Genes Dis, 2021. **8**(4): p. 463-474.
24. Patil, G. and S. Li, *Tripartite motif proteins: an emerging antiviral protein family.* Future Virol, 2019. **14**(2): p. 107-122.
25. Zhang, Y., et al., *The roles and targeting options of TRIM family proteins in tumor.* Front Pharmacol, 2022. **13**: p. 999380.
26. Jourdain, A.A., et al., *The FASTK family of proteins: emerging regulators of mitochondrial RNA biology.* Nucleic Acids Res, 2017. **45**(19): p. 10941-10947.
27. de Azevedo, P.G., et al., *Whole-exome identifies germline variants in families with obstructive sleep apnea syndrome.* Front Genet, 2023. **14**: p. 1137817.
28. Lebrero-Fernandez, C., et al., *Altered expression of Butyrophilin (BTN) and BTN-like (BTNL) genes in intestinal inflammation and colon cancer.* Immun Inflamm Dis, 2016. **4**(2): p. 191-200.
29. Lu, Z., et al., *Mouse myosin-19 is a plus-end-directed, high-duty ratio molecular motor.* J Biol Chem, 2014. **289**(26): p. 18535-48.
30. Shi, P., et al., *Mechanical instability generated by Myosin 19 contributes to mitochondria cristae architecture and OXPHOS.* Nat Commun, 2022. **13**(1): p. 2673.
31. Broday, L., Y.W. Lee, and M. Costa, *5-azacytidine induces transgene silencing by DNA methylation in Chinese hamster cells.* Mol Cell Biol, 1999. **19**(4): p. 3198-204.
32. Moessinger, C., et al., *Human lysophosphatidylcholine acyltransferases 1 and 2 are located in lipid droplets where they catalyze the formation of phosphatidylcholine.* J Biol Chem, 2011. **286**(24): p. 21330-9.

33. Wang, Q., et al., *A protein-encoding CCDC7 circular RNA inhibits the progression of prostate cancer by up-regulating FLRT3.* NPJ Precis Oncol, 2024. **8**(1): p. 11.
34. Yamaguchi, M., et al., *Anthrax toxin receptor 2 is a potential therapeutic target for non-small cell lung carcinoma with MET exon 14 skipping mutations.* Exp Cell Res, 2022. **413**(2): p. 113078.
35. Philp, L.K., et al., *SGTA: a new player in the molecular co-chaperone game.* Horm Cancer, 2013. **4**(6): p. 343-57.
36. Gordon-Lipkin, E.M., et al., *Primary oxidative phosphorylation defects lead to perturbations in the human B cell repertoire.* Front Immunol, 2023. **14**: p. 1142634.
37. Petrosino, J.M., et al., *CCN2 participates in overload-induced skeletal muscle hypertrophy.* Matrix Biol, 2022. **106**: p. 1-11.
38. Leiblich, A., et al., *Lactate dehydrogenase-B is silenced by promoter hypermethylation in human prostate cancer.* Oncogene, 2006. **25**(20): p. 2953-60.
39. Zhang, X.D., et al., *Clinical implications of RAB13 expression in pan-cancer based on multi-databases integrative analysis.* Sci Rep, 2023. **13**(1): p. 16859.
40. Mauldin, J.P., et al., *A link between the cytoplasmic engulfment protein Elmo1 and the Mediator complex subunit Med31.* Curr Biol, 2013. **23**(2): p. 162-7.
41. Stiles, A.R. and D.W. Russell, *SRD5A3: A surprising role in glycosylation.* Cell, 2010. **142**(2): p. 196-8.
42. Narumi, S., et al., *GWAS of thyroid dysgenesis identifies a risk locus at 2q33.3 linked to regulation of Wnt signaling.* Hum Mol Genet, 2022. **31**(23): p. 3967-3974.
43. Wolfson, R.L., et al., *KICSTOR recruits GATOR1 to the lysosome and is necessary for nutrients to regulate mTORC1.* Nature, 2017. **543**(7645): p. 438-442.
44. Diana, P. and G.M.G. Carvalheira, *NIBAN1, Exploring its Roles in Cell Survival Under Stress Context.* Front Cell Dev Biol, 2022. **10**: p. 867003.
45. Gomes, C. and J. Soh, *DnaJC18, a Novel Type III DnaJ Family Protein, is Expressed Specifically in Rat Male Germ Cells.* Dev Reprod, 2017. **21**(3): p. 237-247.
