# Supplementary material for: Skeletal Muscle mRNA Splicing Variants Association With Four Different Fitness and Energetic Measures in the GESTALT Study
Source: J Cachexia Sarcopenia Muscle. 2024 Dec 2;16(1):e13603. doi: 10.1002/jcsm.13603 (PMC11695105; doi:10.1002/jcsm.13603)
Supplement: Supplementary file 1 — Supplementary materials. [file JCSM-16-e13603-s001.zip › S9_Supplementary Figure S9.pdf]

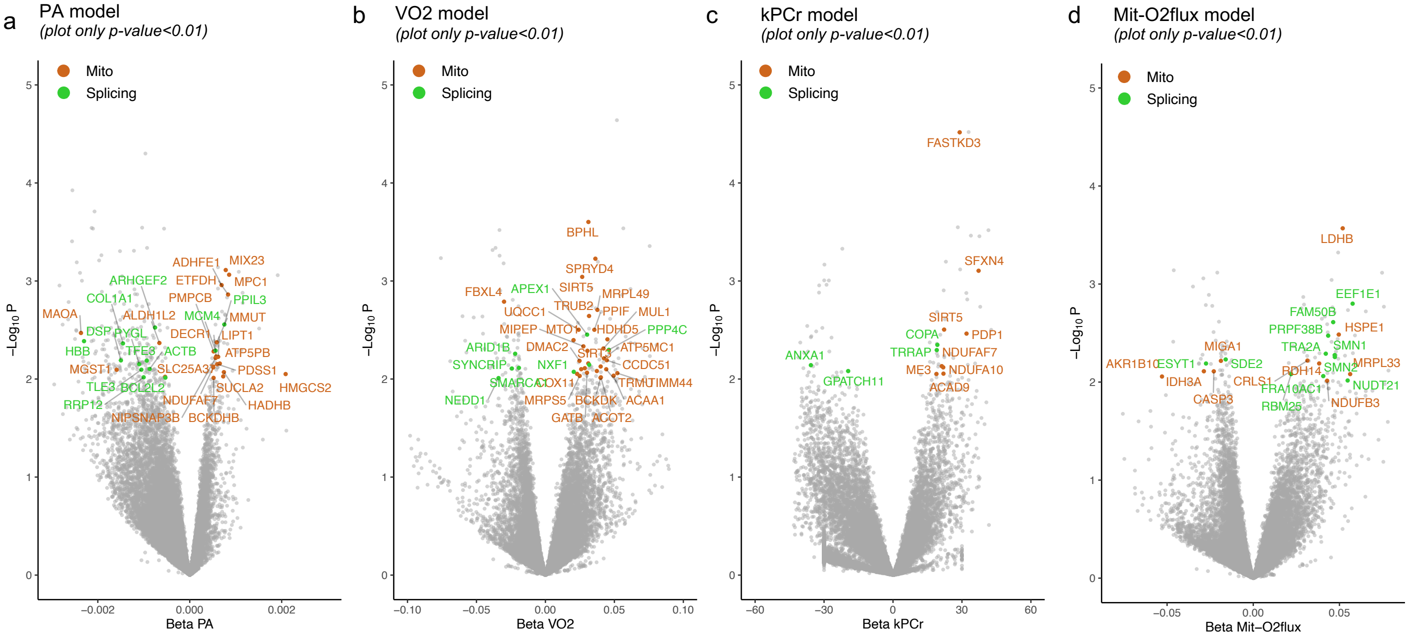
a
PA model
(plot only p-value<0.01)
b
VO2 model
(plot only p-value<0.01)
c
kPCr model
(plot only p-value<0.01)
d
Mit-O2flux model
(plot only p-value<0.01)
Mito
Splicing
Beta PA
Beta VO2
Beta kPCr
Beta Mit−O2flux
−Log10 P
FASTKD3
SFXN4
LDHB
BPHL
SPRYD4
EEF1E1
HSPE1
MRPL33
NUDT21
NDUFB3
HMGCS2
HADHB
