# Supplementary material for: Skeletal Muscle mRNA Splicing Variants Association With Four Different Fitness and Energetic Measures in the GESTALT Study
Source: J Cachexia Sarcopenia Muscle. 2024 Dec 2;16(1):e13603. doi: 10.1002/jcsm.13603 (PMC11695105; doi:10.1002/jcsm.13603)
Supplement: Supplementary file 1 — Supplementary materials. [file JCSM-16-e13603-s001.zip › S10_Supplementary Table S10.pdf]

S10

Model	Beta	Gene	Function (mitochondria)	Reference
**PA**	Up	*MIX23*	mitochondrial protein import machinery	[1]
	Up	*MPC1*	mitochondrial pyruvate carrier	[2, 3]
	Up	*ADHFE1*	adipocyte function and energy metabolism	[4]
**VO2**	Up	*BPHL*	human serine hydrolase	[5]
	Up	*SPRYD4*	unknown mitochondrial function	
	Up	*SIRT5*	mitochondrial metabolism regulation (and others)	[6]
**kPCr**	Up	*FASTKD3*	energy balance of mitochondria under stress	[7]
	Up	*SFXN4*	mitochondrial respiration	[8]
	Up	*SIRT5*	mitochondrial metabolism regulation (and others)	[6]
**MitO2flux**	Up	*LDHB*	lactate-to-pyruvate conversion	[9]
	Up	*HSPE1*	mitochondrial morphology	[10]
	Up	*CRLS1*	mitochondrial membrane integrity	[11]

Table S10: Top three significant ($p<0.01$) mitochondria-related [12] protein-coding mRNAs (genes) for each of the four energetic measurements performed. Model, beta, gene name, function and reference from literature is provided

1. Zoller, E., et al., The intermembrane space protein Mix23 is a novel stress-induced mitochondrial import factor. J Biol Chem, 2020. 295(43): p. 14686-14697.
2. Bricker, D.K., et al., A mitochondrial pyruvate carrier required for pyruvate uptake in yeast, Drosophila, and humans. Science, 2012. 337(6090): p. 96-100.
3. Herzig, S., et al., Identification and functional expression of the mitochondrial pyruvate carrier. Science, 2012. 337(6090): p. 93-6.
4. Chen, Q., Q. Wu, and Y. Peng, ADHFE1 is a correlative factor of patient survival in cancer. Open Life Sci, 2021. 16(1): p. 571-582.
5. Hu, Y., et al., Effect of biphenyl hydrolase-like (BPHL) gene disruption on the intestinal stability, permeability and absorption of valacyclovir in wildtype and Bphl knockout mice. Biochem Pharmacol, 2018. 156: p. 147-156.
6. Ji, Z., G.H. Liu, and J. Qu, Mitochondrial sirtuins, metabolism, and aging. J Genet Genomics, 2022. 49(4): p. 287-298.
7. Jourdain, A.A., et al., The FASTK family of proteins: emerging regulators of mitochondrial RNA biology. Nucleic Acids Res, 2017. 45(19): p. 10941-10947.
8. Paul, B.T., et al., Sideroflexin 4 affects Fe-S cluster biogenesis, iron metabolism, mitochondrial respiration and heme biosynthetic enzymes. Sci Rep, 2019. 9(1): p. 19634.

9. Chen, Y.J., et al., Lactate metabolism is associated with mammalian mitochondria. Nat Chem Biol, 2016. 12(11): p. 937-943.
10. Yeung, N., et al., Role of human HSPE1 for OPA1 processing independent of HSPD1. iScience, 2023. 26(2): p. 106067.
11. Lee, R.G., et al., Deleterious variants in CRLS1 lead to cardiolipin deficiency and cause an autosomal recessive multi-system mitochondrial disease. Hum Mol Genet, 2022. 31(21): p. 3597-3612.
12. Rath, S., et al., MitoCarta3.0: an updated mitochondrial proteome now with sub-organelle localization and pathway annotations. Nucleic Acids Res, 2021. 49(D1): p. D1541-D1547.
