# Supplementary material for: Skeletal Muscle mRNA Splicing Variants Association With Four Different Fitness and Energetic Measures in the GESTALT Study
Source: J Cachexia Sarcopenia Muscle. 2024 Dec 2;16(1):e13603. doi: 10.1002/jcsm.13603 (PMC11695105; doi:10.1002/jcsm.13603)
Supplement: Supplementary file 1 — Supplementary materials. [file JCSM-16-e13603-s001.zip › S11_Supplementary Table S11.pdf]

S11

Model	Beta	Gene	Function (splicing)	Reference
**PA**	Up	*MCM4*	pre-replication complex	[1]
	Up	*PPIL3*	chromatin modification, transcription, mRNA splicing	[2]
	Down	*ARHGEF2*	enzyme regulators spliced variants	[3]
**VO2**	Up	*APEX1*	alternative splicing regulator	[4]
	Up	*PPP4C*	serine/threonine phosphatase	[5]
	Down	*ARIDB1*	transcription and splicing regulation	[6]
**kPCr**	Up	*COPA*	Golgi complex to the endoplasmic reticulum	[7]
	Up	*TRRAP*	epigenetic transcription activation	[8]
	Down	*ANXA1*	multiple cellular processes regulation	[9]
**MitO2flux**	Up	*EEF1E1*	Immune infiltration, cancer-related	[10]
	Up	*FAM50B*	splicing factor in cancer	[11]
	Up	*PRPF38B*	pre mRNA splicing factor	[12]

Table S11: Top three significant ($p<0.01$) splicing-related [13] protein-coding mRNAs (genes) for each of the four energetic measurements performed. Model, beta, gene name, function and reference from literature is provided

1. Tan, Y., L. Ding, and G. Li, *MCM4 acts as a biomarker for LUAD prognosis.* J Cell Mol Med, 2023. **27**(21): p. 3354-3362.
2. Rajiv, C. and T.L. Davis, *Structural and Functional Insights into Human Nuclear Cyclophilins.* Biomolecules, 2018. **8**(4).
3. Chen, H., et al., *Long-Read RNA Sequencing Identifies Alternative Splice Variants in Hepatocellular Carcinoma and Tumor-Specific Isoforms.* Hepatology, 2019. **70**(3): p. 1011-1025.
4. Peng, L., et al., *APEX1 regulates alternative splicing of key tumorigenesis genes in non-small-cell lung cancer.* BMC Med Genomics, 2022. **15**(1): p. 147.
5. Cohen, P.T., A. Philp, and C. Vazquez-Martin, *Protein phosphatase 4--from obscurity to vital functions.* FEBS Lett, 2005. **579**(15): p. 3278-86.
6. Reddy, D., et al., *Paraspeckles interact with SWI/SNF subunit ARID1B to regulate transcription and splicing.* EMBO Rep, 2023. **24**(1): p. e55345.
7. Vece, T.J., et al., *Copa Syndrome: a Novel Autosomal Dominant Immune Dysregulatory Disease.* J Clin Immunol, 2016. **36**(4): p. 377-387.
8. Detilleux, D., et al., *The TRRAP transcription cofactor represses interferon-stimulated genes in colorectal cancer cells.* Elife, 2022. **11**.
9. Buckingham, J.C., et al., *Annexin 1, glucocorticoids, and the neuroendocrine-immune interface.* Ann N Y Acad Sci, 2006. **1088**: p. 396-409.

10. Han, R., et al., *A Novel HCC Prognosis Predictor EEF1E1 Is Related to Immune Infiltration and May Be Involved in EEF1E1/ATM/p53 Signaling.* Front Oncol, 2021. **11**: p. 700972.
11. Ding, Q., et al., *Identification of the prognostic signature based on genomic instability-related alternative splicing in colorectal cancer and its regulatory network.* Front Bioeng Biotechnol, 2022. **10**: p. 841034.
12. Abdel-Fatah, T.M.A., et al., *The localization of pre mRNA splicing factor PRPF38B is a novel prognostic biomarker that may predict survival benefit of trastuzumab in patients with breast cancer overexpressing HER2.* Oncotarget, 2017. **8**(68): p. 112245-112257.
13. Cvitkovic, I. and M.S. Jurica, *Spliceosome database: a tool for tracking components of the spliceosome.* Nucleic Acids Res, 2013. **41**(Database issue): p. D132-41.
