# Supplementary material for: Skeletal Muscle mRNA Splicing Variants Association With Four Different Fitness and Energetic Measures in the GESTALT Study
Source: J Cachexia Sarcopenia Muscle. 2024 Dec 2;16(1):e13603. doi: 10.1002/jcsm.13603 (PMC11695105; doi:10.1002/jcsm.13603)
Supplement: Supplementary file 1 — Supplementary materials. [file JCSM-16-e13603-s001.zip › S12_Supplementary Table S12.pdf]

S12

Model shared	Beta	Gene	Function	Reference
**PA, kPCr**	Up	*NDUFAF7*	complex I NADH dehydrogenase, ubiquinone	[1, 2]
**PA, kPCr**	Up	*GOT1*	TCA cycle, energy metabolism, ROS balance	[3, 4]
**VO2, kPCr**	Up	*SIRT5*	mitochondrial metabolism regulation, others	[5-7]
**VO2, kPCr**	Up	*ZNF366*	transcriptional repression activity	[8]
**PA, VO2**	Down	*CX3CR1*	macrophage phagocytosis, muscle injury repair	[9]
**VO2, kPCr**	Down	*AZI2*	inhibit DNA methylation	[10]
**PA, kPCr**	Down	*SGTB*	co-chaperone function, regulates ATPase activity	[11]

Table S12: Shared significant ($p<0.01$) protein-coding mRNAs (genes) in at least two of the four energetic measurements performed. Models shared, beta, gene name, function and reference from literature is provided

1. Rhein, V.F., et al., *NDUFAF7 methylates arginine 85 in the NDUFS2 subunit of human complex I.* J Biol Chem, 2013. 288(46): p. 33016-26.
2. Zurita Rendon, O., et al., *The arginine methyltransferase NDUFAF7 is essential for complex I assembly and early vertebrate embryogenesis.* Hum Mol Genet, 2014. 23(19): p. 5159-70.
3. Altman, B.J., Z.E. Stine, and C.V. Dang, *From Krebs to clinic: glutamine metabolism to cancer therapy.* Nat Rev Cancer, 2016. 16(10): p. 619-34.
4. Xu, W., et al., *GOT1 regulates CD8(+) effector and memory T cell generation.* Cell Rep, 2023. 42(1): p. 111987.
5. Fabbrizi, E., et al., *Emerging Roles of SIRT5 in Metabolism, Cancer, and SARS-CoV-2 Infection.* Cells, 2023. 12(6).
6. Zhu, S., et al., *Sirt5 Deficiency Causes Posttranslational Protein Malonylation and Dysregulated Cellular Metabolism in Chondrocytes Under Obesity Conditions.* Cartilage, 2021. 13(2_suppl): p. 1185S-1199S.
7. Ji, Z., G.H. Liu, and J. Qu, *Mitochondrial sirtuins, metabolism, and aging.* J Genet Genomics, 2022. 49(4): p. 287-298.
8. Lopez-Garcia, J., et al., *ZNF366 is an estrogen receptor corepressor that acts through CtBP and histone deacetylases.* Nucleic Acids Res, 2006. 34(21): p. 6126-36.
9. Zhao, W., et al., *CX3CR1 deficiency delays acute skeletal muscle injury repair by impairing macrophage functions.* FASEB J, 2016. 30(1): p. 380-93.
10. Broday, L., Y.W. Lee, and M. Costa, *5-azacytidine induces transgene silencing by DNA methylation in Chinese hamster cells.* Mol Cell Biol, 1999. 19(4): p. 3198-204.

11. Philp, L.K., et al., *SGTA: a new player in the molecular co-chaperone game.* Horm Cancer, 2013. 4(6): p. 343-57.
