# Supplementary material for: Skeletal Muscle mRNA Splicing Variants Association With Four Different Fitness and Energetic Measures in the GESTALT Study
Source: J Cachexia Sarcopenia Muscle. 2024 Dec 2;16(1):e13603. doi: 10.1002/jcsm.13603 (PMC11695105; doi:10.1002/jcsm.13603)
Supplement: Supplementary file 1 — Supplementary materials. [file JCSM-16-e13603-s001.zip › S13_Supplementary Table S13.pdf]

S13

Model shared	Gene	Function	Reference
PA, VO2, kPCr, MitO2flux	*AOPEP*	muscle protein metabolism and neural maintenance	[1, 2]
PA, VO2, kPCr	*RPS6KB1*	cell cycle, growth and survival	[3]
PA, VO2, kPCr	*ZEB1*	epithelial-mesenchymal transition	[4]
PA, VO2, kPCr	*BANF1*	DNA repair	[5]
PA, kPCr, MitO2flux	*PCBP-AS1*	alternative splicing	[6]
PA, kPCr, MitO2flux	*MAPK10*	muscle size, hypertrophy, regeneration	[7, 8]
PA, VO2, kPCr	*NDUFS8*	mitochondrial formation, muscle metabolism	[9, 10]
PA, VO2, kPCr	*PRKN*	mitophagy mediator	[11]
PA, VO2, kPCr	*KIAA1109*	neuromuscular junction formation	[12]
PA, VO2, kPCr	*XPC*	nucleotide/DNA excision repair	[13]
PA, VO2, kPCr	*MYBPC1*	slow-type skeletal muscle function	[14]

Table S13: Shared significant (p<0.01) alternative splicing events by protein-coding mRNAs (gene family name) in at least three of the four energetic measurements performed. Models shared, gene name, function and reference from literature is provided

1. Chen, Y., et al., *Structure of the transcribing RNA polymerase II-Elongin complex.* Nat Struct Mol Biol, 2023. 30(12): p. 1925-1935.
2. Zech, M., et al., *Biallelic AOPEP Loss-of-Function Variants Cause Progressive Dystonia with Prominent Limb Involvement.* Mov Disord, 2022. 37(1): p. 137-147.
3. Bahrami, B.F., et al., *p70 Ribosomal protein S6 kinase (Rps6kb1): an update.* J Clin Pathol, 2014. 67(12): p. 1019-25.
4. Zhang, P., Y. Sun, and L. Ma, *ZEB1: at the crossroads of epithelial-mesenchymal transition, metastasis and therapy resistance.* Cell Cycle, 2015. 14(4): p. 481-7.
5. Burgess, J.T., et al., *Barrier-to-autointegration-factor (Banf1) modulates DNA double-strand break repair pathway choice via regulation of DNA-dependent kinase (DNA-PK) activity.* Nucleic Acids Res, 2021. 49(6): p. 3294-3307.
6. Huang, S., et al., *PCBP1 regulates the transcription and alternative splicing of metastasis-related genes and pathways in hepatocellular carcinoma.* Sci Rep, 2021. 11(1): p. 23356.
7. Thomson, D.M., *The Role of AMPK in the Regulation of Skeletal Muscle Size, Hypertrophy, and Regeneration.* Int J Mol Sci, 2018. 19(10).
8. Yuan, J., et al., *The MAPK and AMPK signalings: interplay and implication in targeted cancer therapy.* J Hematol Oncol, 2020. 13(1): p. 113.

9. Motohashi, N., K. Minegishi, and Y. Aoki, *Inherited myogenic abilities in muscle precursor cells defined by the mitochondrial complex I-encoding protein.* Cell Death Dis, 2023. 14(10): p. 689.
10. Wang, S., et al., *Emerging Roles of NDUFS8 Located in Mitochondrial Complex I in Different Diseases.* Molecules, 2022. 27(24).
11. Chatzinikita, E., et al., *The Role of Mitophagy in Skeletal Muscle Damage and Regeneration.* Cells, 2023. 12(5).
12. Liu, Y. and W. Lin, *KIAA1109 is required for survival and for normal development and function of the neuromuscular junction in mice.* bioRxiv, 2022.
13. D'Errico, M., et al., *New functions of XPC in the protection of human skin cells from oxidative damage.* EMBO J, 2006. 25(18): p. 4305-15.
14. Heling, L., M.A. Geeves, and N.M. Kad, *MyBP-C: one protein to govern them all.* J Muscle Res Cell Motil, 2020. 41(1): p. 91-101.
