# Supplementary figures and images for: Skeletal Muscle mRNA Splicing Variants Association With Four Different Fitness and Energetic Measures in the GESTALT Study
Source: J Cachexia Sarcopenia Muscle. 2024 Dec 2;16(1):e13603. doi: 10.1002/jcsm.13603 (PMC11695105; doi:10.1002/jcsm.13603)

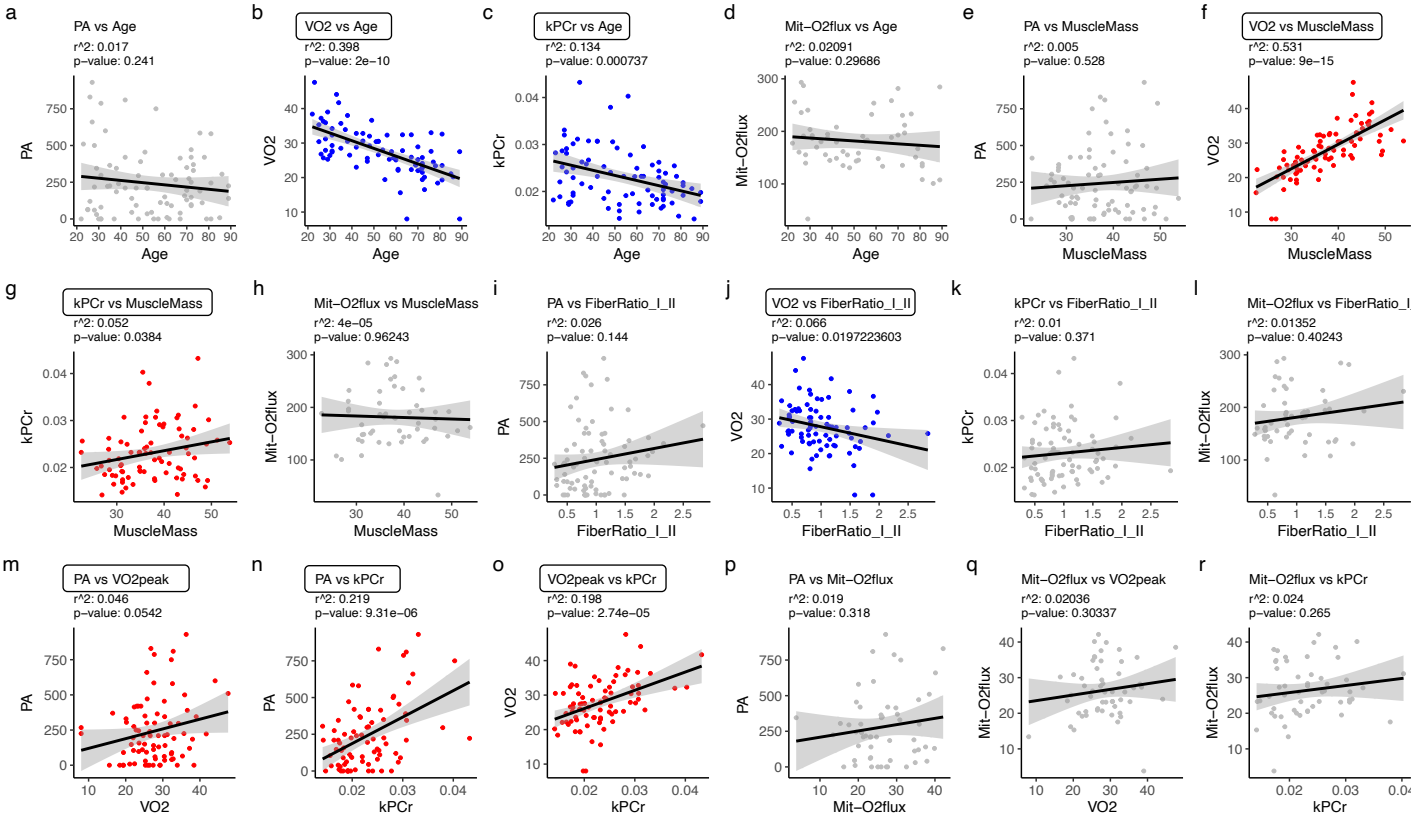

Supplement: Supplementary file 1 — Supplementary materials. [file JCSM-16-e13603-s001.zip › S5_Supplementary Figure S5.pdf]

**a** Splicing events ~ Aging  
Top 20 (by p-adj) each cohort

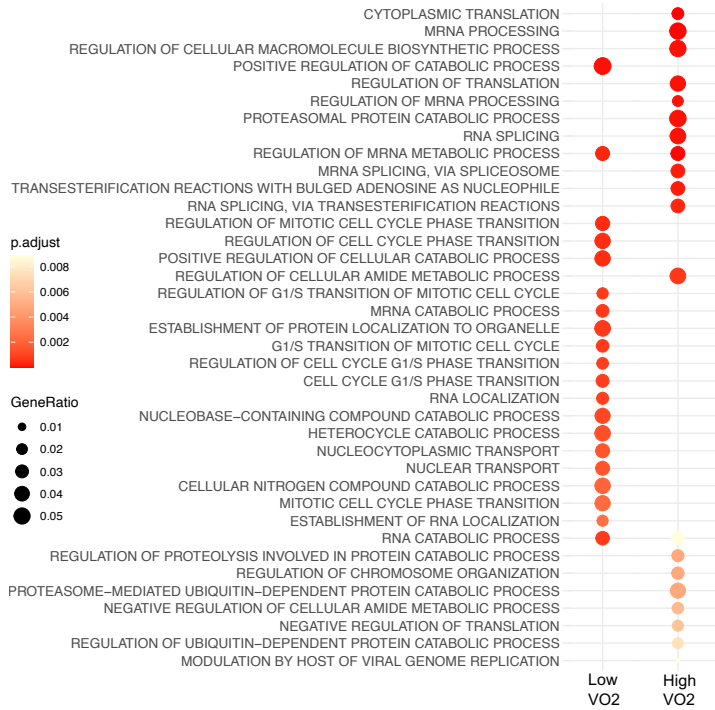

**b** Splicing events ~ Aging  
Shared among Top 100 (by p-adj)

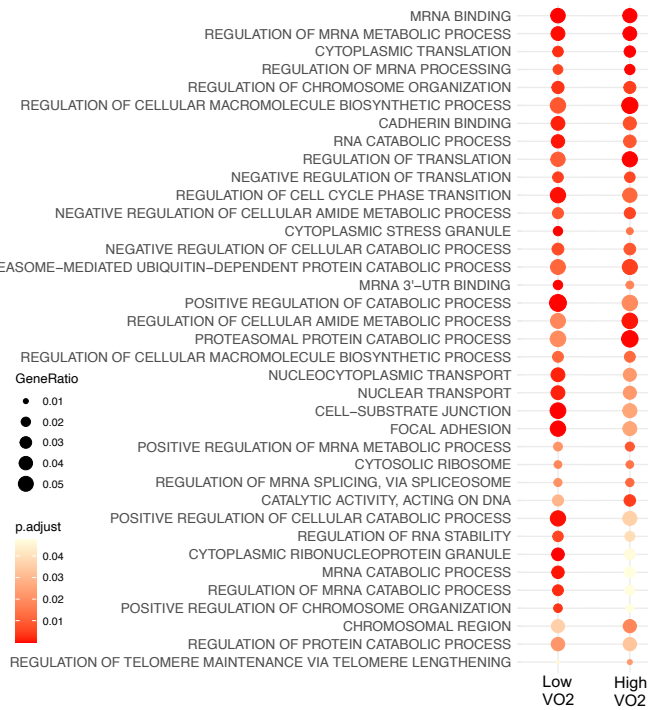

Supplement: Supplementary file 1 — Supplementary materials. [file JCSM-16-e13603-s001.zip › S14_Supplementary Figure S14.pdf]
